# Plasma L-Ergothioneine Measurement by High-Performance Liquid Chromatography and Capillary Electrophoresis after a Pre-Column Derivatization with 5-Iodoacetamidofluorescein (5-IAF) and Fluorescence Detection

**DOI:** 10.1371/journal.pone.0070374

**Published:** 2013-07-29

**Authors:** Salvatore Sotgia, Elisabetta Pisanu, Gianfranco Pintus, Gian Luca Erre, Gerard Aime Pinna, Luca Deiana, Ciriaco Carru, Angelo Zinellu

**Affiliations:** 1 Department of Biomedical Sciences, University of Sassari, Sassari, Italy; 2 Department of Clinical and Experimental Medicine, University of Sassari, Sassari, Italy; 3 Department of Chemistry and Medicinal Chemistry, University of Sassari, Sassari, Italy; University of Helsinki, Finland

## Abstract

Two sensitive and reproducible capillary electrophoresis and high-performance liquid chromatography-fluorescence procedures were established for quantitative determination of L-egothioneine in plasma. After derivatization of L-ergothioneine with 5-iodoacetamidofluorescein, the separation was carried out by HPLC on an ODS-2 C-18 sperisorb column by using a linear gradient elution and by HPCE on an uncoated fused silica capillary, 50 µm id, and 60 cm length. The methods were validated and found to be linear in the range of 0.3 to 10 µmol/l. The limit of quantification was 0.27 µmol/l for HPCE and 0.15 µmol/l for HPLC. The variations for intra- and inter-assay precision were around 6 RSD%, and the mean recovery accuracy close to 100% (96.11%).

## Introduction

L-Ergothioneine (ERT; 2-mercaptohistidine trimethylbetaine) is an unusual sulphur-containing amino acid widely distributed in higher plants and in the organs of several animals. ERT is exclusively synthesized in some fungi and bacteria from the precursors cysteine, methionine, and histidine [1]. In mammals ERT is acquired exclusively through dietary means and accumulated in cells and tissues normally exposed to oxidative stress and involved in inflammatory response [2]. The physiological functions so far suggested for ERT span a wide range and include antioxidant and scavenging activities [3], protection against ischemia/reperfusion-induced injury [4], regulation of metalloenzymes [5], inhibition of DNA oxidation by peroxynitrite (ONOO^−^) in the human neuronal hybridoma cell line [3], catalysis of carboxylation or decarboxylation reactions [6], neuroprotection against NMDA excitotoxicity in rats, transport of cations or carbon dioxide [7], neuroprotection against cisplatin toxicity in mice [8], and mediation of thyroid or anticholinergic action [6]. ERT also chelates redox-active bivalent cations such as copper and zinc [9], [10], prevents the pro-oxidant effects of copper [11], and shows radioprotective activity even at low concentrations [12]. In a pathological context, unexpected high levels of ERT have been observed in the blood of patients affected by autoimmune disorders such as rheumatoid arthritis and Crohn's disease [13], [14]. However, despite the efforts, the major biological role of ERT has not been clarified yet. Analytical procedures for evaluation of ERT contents [15]–[20] have been developed since its discovery but none of these assays have proved to be suitable for a convenient general use as they have long runtime or it is difficult to obtain analytical equipment. Moreover, these assay methods have been principally developed to determine higher concentration of ERT and have proved to be not sensitive enough to measure the low levels of endogenous ERT found in some matrices, such as, e.g., plasma. As a result, the analysis of ERT in biological matrices other than whole blood has been hampered by the lack of analytical methods with adequate sensitiveness. To measure ERT in whole blood, we have recently developed a method that exploits the ability of HILIC technique to increase the retention and the selectivity of polar solutes [21], [22] in combination with an ultra-performance liquid chromatography. As expected, however, this method [23] was unable to detect ERT in human plasma. In order to increase sensitivity and performance of the analysis, in this work we report both a rapid high-performance liquid chromatography and a capillary electrophoresis (HPCE) method, for the quantitative assay of the small amount of plasma ERT based on a selective pre-column derivatization with 5-iodoacetamidofluorescein (5-IAF) and fluorescence detection.

## Materials and Methods

### Chemicals

Acetonitrile (ACN) HPLC grade was purchased from Nova Chimica srl (Milan, Italy) while methanol (MeOH) for HPLC from Carlo Erba Reagents (Milan, Italy). Potassium phosphate dibasic, sodium phosphate tribasic dodecahydrate, DMSO, and 5-iodoacetamidofluorescein (5-IAF) were obtained from Sigma Aldrich Italia (Milan, Italy) while L-ergothioneine (ERT) from DBA Italia srl (Milan, Italy). High-purity water was used throughout the experiments and it was obtained by a Millipore Milli-Q system.

### Solutions

A standard solution of L-ergothioneine was prepared in ultrapure water as a 4.36 mmol/l stock solution and stored at −20°C until use. Fresh working standard solutions were prepared by diluting the stock solution with ultrapure water to the required concentrations before use. 5-IAF was prepared in DMSO as a 20 mmol/l stock solution and stored at −20°C until use. Fresh working 5-IAF solution was obtained by diluting the stock solution to 770 µmol/l with a freshly daily 150 mmol/l sodium phosphate tribasic dodecahydrate solution at pH 13.

### Sample Treatment

To precipitate the proteins, a 100 µl-volume of ACN was added to 100 µl of sample and mixed thoroughly by vigorous vortex-mixing. After centrifugation at 17000×g for 10 min at room temperature, 50 µl of the 5-IAF working solution were added to 150 µl of supernatant and, after vigorous vortex-mixing, reaction mixture was left in a light-protected area for 30 min at room temperature. Finally, for the HPLC and HPCE analysis, samples were diluted with water two and fifty times respectively.

### Participants to Study and Sample Collection

The samples for this study were derived from our previous work [24], by simple random sampling of the subjects belonging to the control cohort. In particular, were randomly selected thirty-four apparently healthy volunteers (16 males, 18 females) aged from 66 to 94. After informed, written consent was obtained, then whole blood was collected by venipuncture in 5.4 mg K3EDTA vacutainer tubes. Without delay, it was centrifuged at 4° and 3000 g for 10 minutes to separate plasma, which was stored for five years at −80°C before analysis. The study was performed according to the Declaration of Helsinki and all procedures were approved by the ethics committee of Azienda USL n°1 - Sassari (710/2/L 2008).

### HPLC Equipment and Chromatographic Conditions

The LC system consisted of a Waters system model Alliance 2695 equipped with a Waters 474 fluorescence detector. The separation was achieved on a 250 mm×4.6 mm Waters C18 5 µm spherisorb ODS2 column by using two eluents composed of a mixture of 100 mmol/l potassium phosphate dibasic/ACN/MeOH/water (40∶7:3∶50, v/v/v/v) (Eluent A) and of a mixture of a mixture of 100 mmol/l potassium phosphate dibasic/ACN/MeOH/water (40∶20:3∶37, v/v/v/v) (Eluent B). Both mobile phases were filtered through a disposable 0.22-µm membrane filter (Millipore, Milford, MA, USA) to remove any particulate matter prior to their use. The eluents were delivered to the chromatographic column applying a linear gradient elution starting with Eluent A changing to Eluent B after 6 min and then to Eluent A after 3 min. The elution was continued for 7 min keeping a flow-rate of 1.2 ml min-1 for the entire duration of the running. Separation was carried out in an air-conditioned room at about 25°C and samples were held at 10°C in the autosampler. Amount injected was 2 µl and column eluates were detected by fluorescence detector at an excitation wavelength of 494 nm and emission wavelength of 518 nm with the signal gain set at ×100 scale expansion.

### HPCE Equipment and Electrophoretic Conditions

For the electrophoretic experiments, a HPCE P/ACE MDQ system equipped with a laser-induced fluorescence detector (LIF) was used (Beckman-Coulter, Fullerton, CA, USA). The system was fitted with a 30 kV power supply with a current limit of 300 µA. The analysis was performed in an uncoated fused silica capillary, 50 µm id, and 60 cm length (50 cm to the detection window), injecting 3.87 nl of sample (0.5 psi×5 s). The separation was carried out by using a 20 mmol/l sodium phosphate tribasic dodecahydrate solution as the running buffer, 15°C, and 30 kV (70 µA) at normal polarity. Derivatized samples were held at 10°C in the autosampler and detected by LIF detector. After each run, the capillary was equilibrated with running buffer for 1 min.

## Results and Discussion

**Figure 1:**
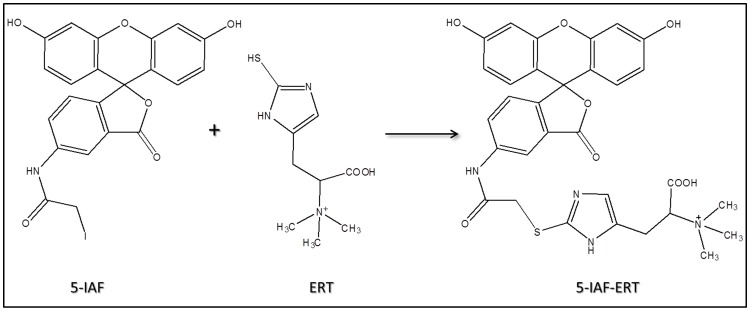
The reaction course of 5-IAF with L-ergothioneine. Iodoacetamide moiety of 5-IAF reacts specifically with -SH group of ERT to yield the stable 5-IAF-ERT thioether which emits green fluorescence with a maximum at 518 nm when it is excited at 494 nm.

**Figure 2:**
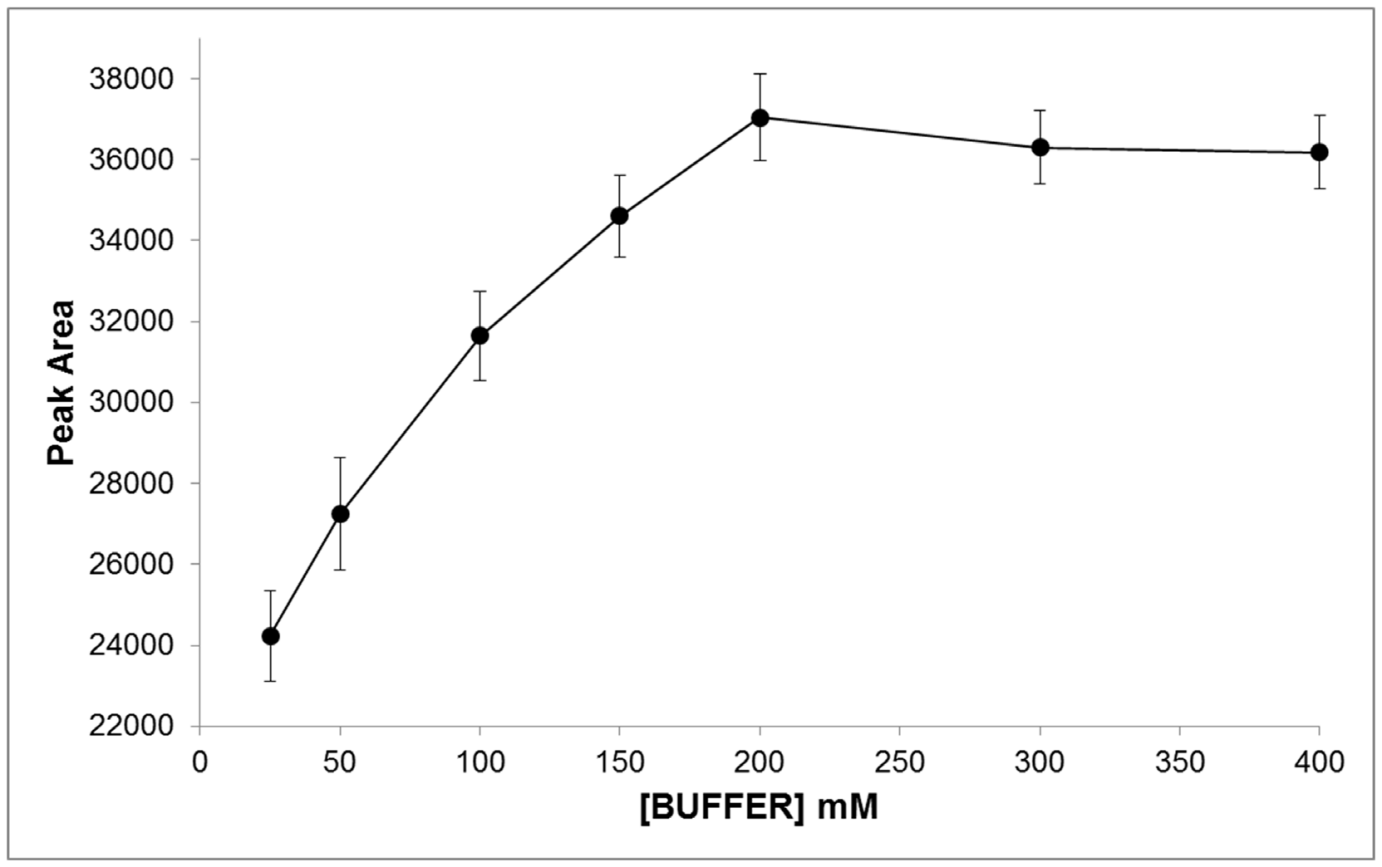
The yield of thioether 5-IAF-ERT as a function of the concentration of the sodium phosphate tribasic dodecahydrate buffer ([BUFFER]) used to prepare the working solution of 5-IAF. The peak area reached a maximum at a buffer concentration of 200 mmol/l, but due to occasional precipitation of the buffer, in the definitive procedure its concentration was fixed in 150 mmol/l.

**Figure 3:**
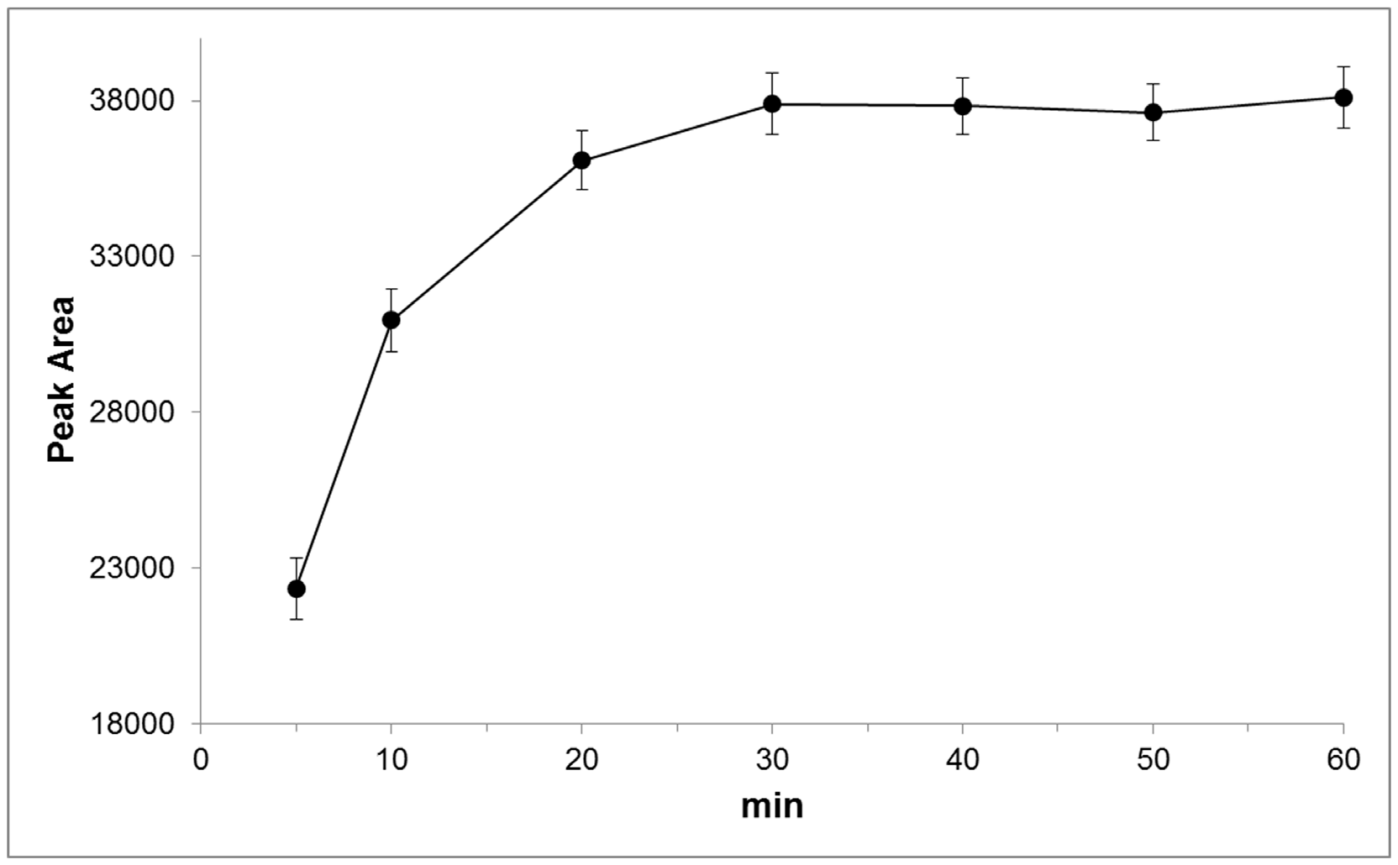
The yield of thioether 5-IAF-ERT as a function of the reaction time. Derivatization conditions were described in the text.

**Figure 4:**
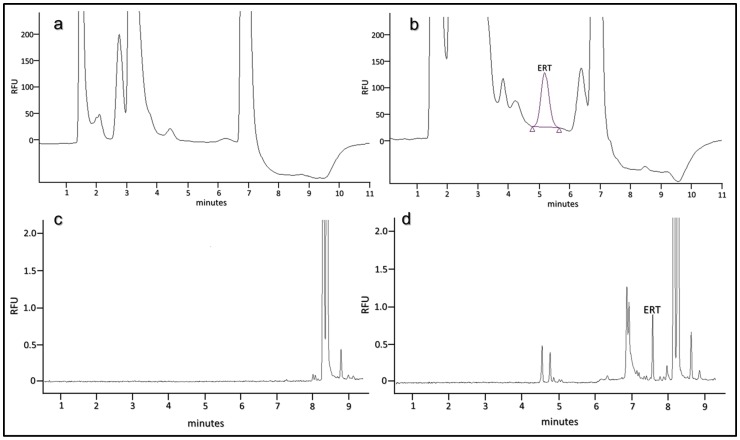
Representative chromatograms of (a) a blank and (b) a real sample and electropherograms of (c) a blank and (d) a real sample. Multiple peaks were mainly due to the unreacted 5-IAF.

**Figure 5:**
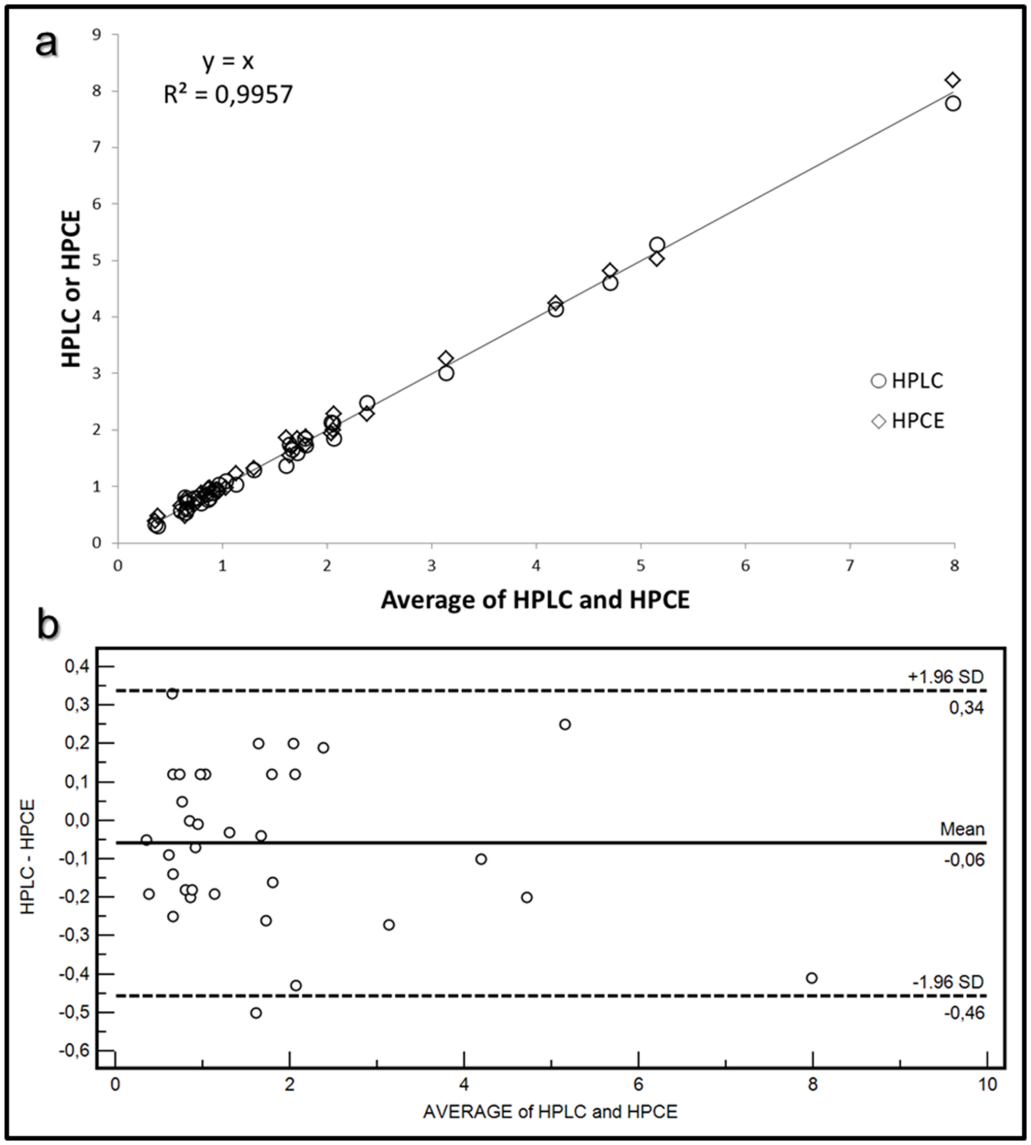
Comparison of data by (a) Sotgia curve and (b) Bland-Altman test. Sotgia regression shows the magnitude difference between the values obtained by HPLC or HPCE from each other and around the line of best fit (average versus average) and provides, as an index of agreement between two methods, a coefficient of determination of 0.9957. Bland–Altman plot shows the differences between the values obtained by HPLC and HPCE method against their averages. Bland–Altman test showed that HPCE method gave, on average, values higher than those of HPLC assays with a bias of −0.06 and limits of agreement ranging between 0.34 and −0.46.

Human plasma contains 2 to 9 fold less ERT than erythrocytes [25], therefore, an improvement of the response in the detection system by derivatization with reagents affording chromophores or fluorophores, is required for its analysis in such matrix. ERT, in fact, has no intrinsic fluorophores and the analytically useful absorption spectrum in the UV range, molar extinction coefficient ε of 1.4×10^−4^ M^−1^ cm^−1^ at 257 nm [2], is not sufficient to allow its detectability at low levels. The chemical structure of ERT reveals that the molecule contains both a carboxyl and a thiol group capable of derivatization. However, taking into account that the reactivity of the carboxylic group is lower than that of the thiol, that for its derivatization, usually, catalysts are needed and that in a biological matrix carboxylic-containing compound are more numerous than thiol, in this work we have used an idoacetamide derivate of fluoresceine, 5-IAF, which possess a thiol-reactive iodoacetyl group at 5-carbon position of the lower ring [26]. This choice also arises from considerations on the tautomerism of ERT. In aqueous solution, in fact, ERT exists as a tautomer between its thiol and thione forms with the last predominating at physiological pH. This peculiar attribute is accountable for the stability and reactivity of ERT compared to other naturally occurring thiols. As thione, in fact, ERT does not react with some sulfhydryl reagent making it difficult its derivatization with common thiol-modifiers such as DTNB. As reported by Carlsson et al, iodoacetamide (IAA), another sulfhydryl-reactive alkylating reagent, is an exception. IAA, as well pH values above 9, are able to destroy the thione character of the ERT by inducing the proton loss from imidazole nitrogen [15]. The use of an idoacetamide derivate such as 5-IAF and of high pH values seemed therefore the most suitable choice to ensure a quantitative derivatization of ERT. Figure 1 shows the course of the reaction of 5-IAF with ERT. 5-IAF was highly reactive with the thiol group and, as already reported in our previous works for the other physiological thiols [27], [28], the reaction proceeded at room temperature in the dark. Figure 2 and 3 display the yield of thioether conjugate (5-IAF-ERT) as a function of the concentration of the sodium phosphate tribasic dodecahydrate buffer used to prepare the working solution of 5-IAF and of the reaction time. The peak area reached a maximum at a buffer concentration of 200 mmol/l (see figure 2) and a plateau over 20 min (see figure 3). However, by using the sodium phosphate tribasic dodecahydrate buffer at the concentration of the maximum reaction yield (200 mmol/l), due to an occasional precipitation of the salt, also a poor reproducibility and random deviations from linearity was obtained. In the definitive procedure, therefore, the concentration of the buffer was fixed in 150 mmol/l and the length of the reaction time in 30 min. 5-IAF concentration was optimized in order to reach the greatest sensitivity quickly and it was substantially the same found in our earlier works. Figure 4 shows both a chromatogram and an electropherogram of a real and of a blank sample. Without interfering peaks, ERT peak was clearly separated from the others in less than 6 (5.2) and 8 (7.5) min in HPLC and HPCE procedures, respectively. Although ERT was eluted from the chromatographic column in fairly short time, to avoid that unreacted 5-IAF continued to elute in subsequent runs, it was necessary to set an elution by gradient. By this way, the total time required for a single run was 11 min and, despite the gradient, from run to run the peak shape was reproducible without distortions as well as retention time and peak area. The calibration plot for ERT showed excellent linearity over a concentration range of 0.3 to 10 µmol/l (R^∧^2 = 0.999) both in the HPLC and HPCE analysis. Intra- and inter-assay precision evaluated by performing 3 replicate analyses of a 0.32, 1.81 and 3.52 µmol/l plasma pool was around 6 RSD% both for HPLC and HPCE analysis. The exact concentrations of these samples (0.32, 1.81 and 4.52 µmol/l) were obtained by multiple addition method and the values were superimposable between HPLC and HPCE method. Recovery experiments conducted to determine the accuracy of the method were performed by spiking 100 µl of a biological sample with 10 µl of standard ERT solutions at three concentration levels (10, 30 and 90 µmol/l). The mean recovery accuracy, calculated as recovery(%) = 100x((C1-C0)/A), where C0 and C1 are determined compound concentrations before and after compound addition, and A is the quantity of compound added, was close to 100% (96.11%). The limit of detection (LOD) and the limit of quantification (LOQ) calculated on three calibration curves following, respectively, the equations 3.3σ/S and 10σ/S, where σ is the standard deviation of the response and S the slope of the calibration plot, were on average around 0.09 and 0.27 µmol/l for HPCE and 0.05 and 0.15 µmol/l for HPLC. The established procedure was applied to the determination of ERT in the enrolled thirty-four volunteers. The average ERT level (mean±SD) in these samples was 1.71±1.60 µmol/l and 1.77±1.64 µmol/l for the HPLC and HPCE analysis, respectively. By using Sotgia test [29] and Bland-Altman test [30], the results obtained by HPLC were compared with those obtained by analyzing the same samples with HPCE. The output of this comparison was displayed in figure 5 and it showed a good agreement among data, ensuring the accuracy of both methods.

### Conclusion

To our knowledge, this is the first work that describes a HPCE assay for the plasma ergothioneine measurement. Derivatization with 5-IAF was also a novelty for the ergothioneine quantification. Both HPLC and HPCE methods were accurate and sensitive enough to measure the small amount of ERT contained in plasma. The pre-column derivatization with 5-IAF was reliable and, together with an easy pre-treatment of the samples, quite fast to allow quick analysis. On the whole, the new assay may be useful for routine analyses, both in the clinical and in the research field, and may help in the understanding of the physiology of this unusual amino acid.
